# Incidence of life-threatening respiratory events after laparoscopic colon surgery with or without continuous respiratory rate monitoring

**DOI:** 10.1186/s40981-017-0127-0

**Published:** 2017-10-13

**Authors:** Hideaki Kawanishi, Junji Egawa, Satoki Inoue, Takashi Shiota, Masahiko Kawaguchi

**Affiliations:** Department of Anesthesiology, Nara Medical University, 840 Shijo-cho, Kashihara, Nara 634-8522 Japan

**Keywords:** Life-threatening respiratory events, Respiratory monitoring, Respiratory depression

## Abstract

**Background:**

Respiratory depression (RD) is a critical complication of general anesthesia. The present study investigated the incidence of postoperative life-threatening respiratory events after laparoscopic colon surgery in patients observed using continuous respiratory rate monitoring [RM; with oxygen saturation by pulse oximetry (SpO_2_)] and traditional respiratory monitoring (TM; SpO_2_ monitoring only). In addition, postoperative incidence rates of RD and desaturation in the RM group were determined.

**Findings:**

In this retrospective observational study, medical records of 214 patients who underwent laparoscopic colon surgery were analyzed. A total of 88 patients with RM were observed and compared with 126 patients with TM. Nineteen patients in the RM group were excluded from the final analyses because of incomplete data. No life-threatening respiratory events were observed in the RM group (0/69), whereas two such events (2/126) occurred in the TM group. Incidence rates of postoperative RD and desaturation within 8 h after surgery were 17.1% (12/69) and 24.3% (17/69), respectively, in the RM group.

**Conclusions:**

No postoperative life-threatening respiratory events were observed in the RM group. Furthermore, the incidence rates of RD and desaturation were noted to be relatively high.

## Introduction

Postoperative respiratory depression (RD) is a potentially life-threatening complication of any major surgery. Its etiology of postoperative RD is multifactorial and includes opioids, residual neuromuscular blocking drugs, anesthetics, and obstructive sleep apnea syndrome (OSAS) [1–5]. In general, laparoscopic colon surgery requires longer anesthesia and surgery times compared with open colectomy [6]. Longer anesthesia time requires higher doses of anesthetics, opioids, and neuromuscular blocking drugs. Surgical technique can also be associated with postoperative RD. The Trendelenburg position and abdominal CO_2_ gas insufflation during surgery have been found to result in the increased risk of upper airway edema and elevated airway resistance [7]. A previous study revealed that the detection of hypoventilation by pulse oximetry is reliable only in room air breathing conditions [8]. Thus, the Anesthesia Patient Safety Foundation recommends the continuous postoperative monitoring of SpO_2_ and respiratory rate, particularly when opioids are used for postoperative analgesia [9]. The definition of postoperative RD is not specific and differs among different studies, and this results in the highly variable incidence of RD [10–12]. Although evidence for the incidence of postoperative respiratory depression is inadequate, the risk of severe postoperative RD may be considerably high in patients undergoing laparoscopic colon surgery. This study compared the incidence rates of postoperative life-threatening respiratory events after laparoscopic colon surgery in patients monitored using continuous respiratory rate and oxygen saturation by pulse oximetry (SpO_2_) [respiratory rate monitoring (RM) group] with those monitored using SpO_2_ only [traditional respiratory monitoring (TM) group]. In addition, the incidence rates of RD and desaturation within 8 h after surgery were assessed in the RM group.

## Methods

This retrospective observational study was approved on March 29, 2016, by the Ethics Committee of Nara Medical University Hospital in Kashihara, Japan (study number 1221; principal investigator Junji Egawa).

Medical records of 214 adult patients who underwent laparoscopic colon surgery were assessed. Surgeries were performed by the same surgical team, with the postoperative monitoring of SpO_2_ and electrocardiogram in all patients. In the RM group, SpO_2_ and acoustic RM sensors were attached after extubation in the operating room. Postoperative respiratory rate could not be monitored in patients who had undergone surgery before June 2012 because of the unavailability of the acoustic RM device (Radical-7 with RRa™, Masimo, Irvine, CA, USA) at our institution. Thus, the RM group included data from June 2012 to January 2014, whereas the TM group included data from January 2010 to April 2012. Incidence rates of postoperative life-threatening events in the RM group (*n* = 88) were compared with those in the TM group (*n* = 126). Life-threatening respiratory events were defined as those requiring mask ventilation, intubation, or cardiopulmonary resuscitation.

Respiratory rate and SpO_2_ data, which were collected using the RM device in the RM group, were saved continuously on the server via the SafetyNet™ system (Masimo, Irvine, CA, USA). Data were extracted from the server to a dedicated computer after 8 h of postoperative monitoring. The incidence rate of RD was defined as a respiratory rate of < 8 breaths/min for an accumulated time of > 10 min within 8 h after extubation. SpO_2_ value was recorded as the average of 8 s SpO_2_ value. Desaturation, defined as an SpO_2_ of < 90% within 8 h after extubation, was assessed for all patients in the RM group. However, SpO_2_ and RR in the TM group were manually recorded by caregivers intermittently every 2–3 h after the surgery. Therefore, these data in the TM group were inadequate for comparison with the RM group.

IBM SPSS Statistics version 24.0 (SPSS Inc., Chicago, IL, USA) was used for all analyses. Dichotomous variables were compared using the chi-square test. Depending on data distribution, results were presented as mean ± standard deviation or median with interquartile range (IQR)*. P* values of < 0.05 were considered statistically significant for all analyses.

## Results

Overall, 19 patients in the RM group were excluded from the analysis due to incomplete data. Therefore, 69 patients in the RM group and 126 in the TM group were included in the final analysis. Life-threatening respiratory events were not observed in the RM group (0/69), whereas two such events occurred in the TM group (2/126) (*P* = 0.54). Table 1 shows the detailed information for the two cases of life-threatening respiratory events. All demographic variables except for the prevalence of chronic obstructive pulmonary disease (COPD) and smoking were similar between the RM and TM groups (Table 2). The duration of anesthesia and surgery, total amount of remifentanil used, and intraoperative fluid balance were noted to be significantly different between the RM and TM groups (Table 3). Table 4 shows the postoperative data. The total amount of fentanyl used postoperatively was slightly higher in the RM group. Although significant differences were not observed in 28-day mortality rates between the two groups, the median hospital stay was significantly shorter in the RM group (8 vs. 10 days, *P* < 0.01). Incidence rates of RD and desaturation within 8 h after extubation were 17.3% (12/69) and 24.6% (17/69), respectively.

**Table 1:** Details of the two cases of life-threatening respiratory events

Variable	Case 1	Case 2
Age (year)	71	75
Gender (M/F)	M	M
BMI (kg/m^2^)	22.44	26.82
ASA grade	2	2
Duration of anesthesia (min)	585	778
Anesthetics	Sevoflurane	Sevoflurane
Rocuronium (mg)^a^	230	373
Fentanyl (μg)^a^	500	600
Total fluid balance (mL)	6630	3860
Postoperative analgesia	EPCA	EPCA
Assumed causes of RD	Opioid	Opioid and residual NMB
Required management	CPR	CPR

*BMI* body mass index, *ASA* American Society of Anesthesiologists, *EPCA* epidural patient-controlled analgesia, *NMB* neuromuscular blockade, *RD* respiratory depression, *CPR* cardiopulmonary resuscitation

^a^Total dose used intraoperatively

**Table 2:** Baseline characteristics of study patients

Variable	Respiratory rate monitoring (*n* = 69)	Traditional monitoring (*n* = 126)	*P* value
Age (year)	64.8 ± 12.7	64.3 ± 13.3	0.818
Gender (M/F)	39/30	69/57	0.881
BMI (kg/m^2^)	22.3 ± 3.2	22.9 ± 3.1	0.201
ASA grade (I/II/III/IV)	12/53/4/0	20/100/6/0	0.908
% VC	107.1 ± 20.3	112.6 ± 14.7	0.052
FEV1.0%	76.3 ± 10.8	79.0 ± 11.1	0.103
Smoking	30/69	30/126	0.006
COPD	11/69	3/126	0.001
Active liver disease^a^	11/69	26/126	0.453
Hemodialysis	4/69	4/126	0.457

Data are presented as mean ± standard deviation

*BMI* body mass index, *ASA* American Society of Anesthesiologists, *%VC* % vital capacity, *FEV1.0%* forced expiratory volume 1.0 (sec) %, *COPD* chronic obstructive pulmonary disease, *DM* diabetes mellitus

^a^Aspartate aminotransferase > 30

**Table 3:** Intraoperative data of study patients

Variable	Respiratory rate monitoring (*n* = 69)	Traditional monitoring (*n* = 126)	*P* value
Duration of anesthesia (min)	346 [123–816]	390 [132–933]	0.002
Duration of surgery (min)	265 [75–719]	314 [82–856]	0.001
Anesthetics (Sev/Des/Pro)	60/2/7	113/0/13	0.158
Rocuronium (mg)^a^	100 [40–235]	116 [0–745]	0.091
Remifentanil (μg)^a^	1668 [0–12,730]	885 [0–24,691]	0.020
Fentanyl (μg)^a^	300 [0–1000]	300 [0–2150]	0.966
Transfusion (mL)	0 [0–280]	0 [0–3500]	0.081
Blood loss (mL)	25 [0–883]	18 [0–2700]	0.841
Infusion (mL)	2200 [200–6250]	2775 [345–7400]	< 0.001
Urea (mL)	329 [0–2458]	547 [5–3435]	< 0.001
Total fluid balance (mL)	1764 [125–4532]	2122 [30–6630]	0.001

Data are presented as median [interquartile range]

*Sev* sevoflurane, *Des* desflurane, *Pro* propofol

^a^Total dose used intraoperatively

**Table 4:** Postoperative analgesia and outcomes of study patients

Variable	Respiratory rate monitoring (*n* = 69)	Traditional monitoring (*n* = 126)	*P* value
EPCA	29/69	104/126	< 0.001
IVPCA	26/69	19/126	0.001
Total fentanyl used (μg)	967 [0–6942]	836 [0–3000]	0.045
Life-threatening respiratory events	0/69	2/126	0.54
Incidence of RD	12/69	Not available	
Incidence of desaturation	17/69	Not available	
Mortality	0/69	2/126	0.535
Hospitalization period	8 [1–83]	10 [3–53]	0.001

*EPCA* epidural patient-controlled analgesia, *IVPCA* intravenous patient-controlled analgesia, *RD* respiratory depression

## Discussion

This retrospective observational study revealed two life-threatening respiratory events among the 126 patients observed using TM, whereas the 69 patients observed using respiratory rate and SpO_2_ monitoring did not. The median hospital stay was significantly shorter in the RM than in the TM group (8 vs. 10 days, respectively; *P* < 0.01). As expected, the incidence rates of RD and desaturation were relatively frequent (17.3 and 24.6%, respectively) within 8 h after surgery.

Studies have revealed that patients present abnormal vital signs minutes to hours before the emergence of critical adverse events [13]. Specifically, respiratory rate is one of the most important, albeit often neglected, vital signs [14]. According to the American Society of Anesthesiologists, nearly all opioid-related RD events (97%) were potentially preventable with the utilization of improved monitoring methods [15].

The Radical-7 with RRa™ enables noninvasive, reproducible, and continuous acoustic respiratory rate and SpO_2_ monitoring measurements. Mimoz et al. showed that continuous RM using the acoustic device correlated well with capnometry [16]. At our institution, all medical staff have access to patient data including SpO_2_ and respiratory rate from bedside device screens as well as the central screen at the nurses’ stations. Staff can also monitor alarms via smart devices that are connected to the in-hospital network. Neither of the patients with life-threatening respiratory events had preoperative cardiovascular disease. Intraoperative and postoperative electrocardiogram and blood pressure prior to the event were within normal limits. Thus, we suspected that the life-threatening respiratory events were due to RD. Moreover, we suspected that the life-threatening respiratory events were caused by opioid and opioid with residual neuromuscular blockade because of their rapid recovery without any complications and absence of other reasons for these events. In these types of cases, a continuous respiratory monitoring system such as that used in our study would be useful in the early detection of severe respiratory events.

While the amount of total fentanyl used for patient-controlled anesthesia as well as the rates of COPD and smoking were higher in the RM group, no change in the incidence of life-threatening respiratory events was observed following the implementation of the RM device postoperatively. Several potential reasons could explain this outcome. First, continuous RM may improve the medical staff’s awareness of postoperative RD. Indeed, 67% of nurses who answered the questionnaire after the implementation of the RM system at our institution responded that they paid more attention to potential postoperative RD (data not shown). Second, anesthesiologists may use anesthetic drugs more carefully and observe patients more closely after extubation. Finally, medical staff on the wards could identify abnormal respiratory patterns more expeditiously and intervene at earlier stages of RD.

This study has certain limitations. First, this was a retrospective single-center study. Moreover, patient data in the RM group were taken from a different time period than those in the TM group (June 2012–January 2014 vs. January 2010–April 2012); thus, the potential contribution of changes in background variables, such as surgical improvements over time, could not be excluded. In addition, the incidence of life-threatening respiratory events at our institution may be too low to detect statistical significance; a multicenter cohort study with difference-in-difference analysis is necessary to address this concern [17]. Furthermore, this study did not include data on patient intervention by nurses in response to monitor alarms. Moreover, OSAS is one of the major risk factors associated with postoperative RD [5, 18]. The prevalence of OSAS is estimated to be between 9 and 24%, with approximately 90% of cases remaining undiagnosed [19]. However, the exact number of patients with OSAS could not be determined in this study. Finally, the duration of anesthesia was longer in the TM than in the RM group (390 vs. 346 min, respectively); however, this difference may not be of clinical significance.

## Conclusions

Life-threatening respiratory events were not observed after laparoscopic colon surgery in patients who underwent continuous RM with SpO_2_. The incidence rates of RD and desaturation were relatively frequent within 8 h after surgery. The incidence of life-threatening RD events was low at our institution; therefore, multicenter studies are necessary to clarify the efficacy of continuous respiratory monitoring with SpO_2_.

## Abbreviations

%VC: % vital capacity
ASA: American Society of Anesthesiologists
BMI: Body mass index
COPD: Chronic obstructive pulmonary disease
DM: Diabetes mellitus
EPCA: Epidural patient-controlled analgesia
FEV1.0%: Forced expiratory volume 1.0 (sec) %
IVPCA: Intravenous patient-controlled analgesia
OSAS: Obstructive sleep apnea syndrome
RD: Respiratory depression
RM: Respiratory rate monitoring
TM: Traditional respiratory monitoring
